# Differential Expression of Tyrosine Hydroxylase Protein and Apoptosis-Related Genes in Differentiated and Undifferentiated SH-SY5Y Neuroblastoma Cells Treated with MPP^+^

**DOI:** 10.1155/2015/734703

**Published:** 2015-11-08

**Authors:** Kawinthra Khwanraj, Chareerut Phruksaniyom, Suriyat Madlah, Permphan Dharmasaroja

**Affiliations:** ^1^Department of Anatomy, Faculty of Science, Mahidol University, Bangkok 10400, Thailand; ^2^Department of Pharmacology, Faculty of Dentistry, Mahidol University, Bangkok 10400, Thailand

## Abstract

The human neuroblastoma SH-SY5Y cell line has been used as a dopaminergic cell model for Parkinson's disease research. Whether undifferentiated or differentiated SH-SY5Y cells are more suitable remains controversial. This study aims to evaluate the expression of apoptosis-related mRNAs activated by MPP^+^ and evaluate the differential expression of tyrosine hydroxylase (TH) in undifferentiated and retinoic acid- (RA-) induced differentiated cells. The western blot results showed a gradual decrease in TH in undifferentiated cells and a gradual increase in TH in differentiated cells from days 4 to 10 after cell plating. Immunostaining revealed a gradual increase in TH along with neuritic outgrowth in differentiated cells on days 4 and 7 of RA treatment. For the study on cell susceptibility to MPP^+^ and the expression of apoptosis-related genes, MTT assay showed a decrease in cell viability to approximately 50% requiring 500 and 1000 *μ*M of MPP^+^ for undifferentiated and RA-differentiated cells, respectively. Using real-time RT-PCR, treatment with 500 *μ*M MPP^+^ led to significant increases in the Bax/Bcl-2 ratio, p53, and caspase-3 in undifferentiated cells but was without significance in differentiated cells. In conclusion, differentiated cells may be more suitable, and the shorter duration of RA differentiation may make the SH-SY5Y cell model more accessible.

## 1. Introduction

The well-established human neuroblastoma SH-SY5Y cell line has been used extensively to study dopaminergic neuron-like behavior in response to toxins in research on Parkinson's disease (PD) and neurotoxicity. Upon stimulation, proliferating SH-SY5Y cells can be differentiated into nonproliferating neuronal cells. Retinoic acid (RA) is a common inducing agent used to induce SH-SY5Y cells to become neurons of a dopaminergic phenotype [1]. Both undifferentiated and RA-differentiated SH-SY5Y cells have been widely used as a cellular model of PD. Controversy exists regarding which type of SH-SY5Y cells is most suitable for use in PD research. When exposed to neurotoxins, such as 1-methyl-4-phenyl-pyridinium ion (MPP^+^) and 6-hydroxydopamine (6-OHDA), RA-differentiated cells expressed higher tolerance as shown by the lower rate of the release of lactate dehydrogenase (LDH), cell mortality, and caspase-3 activation, compared to undifferentiated cells, suggesting that undifferentiated cells are more appropriate for studying neurotoxicity or neuroprotection in experimental PD research [2]. In contrast, another study showed that SH-SY5Y cells become significantly more sensitive to 6-OHDA toxicity during the differentiation process as shown by cell mortality assay and GI_50_ values of 6-OHDA, suggesting that RA-differentiated cells represent a more suitable experimental model for studying the pathophysiology of PD [3].

MPP^+^, an inhibitor of mitochondrial complex I, has been widely used as a neurotoxin because it elicits a severe PD-like syndrome. MPP^+^ induces SH-SY5Y cell death through apoptosis. Treatment of undifferentiated and differentiated SH-SY5Y cells with MPP^+^ causes the loss of cell viability, and condensation and fragmentation of nuclei, which is associated with the elevation of the reactive oxygen species (ROS) levels, an increase in the Bax/Bcl-2 ratio, and the activation of caspase-3 [4–11]. MPP^+^ also activates the expression of the p53 tumor suppressor in SH-SY5Y cells [11–13]. In addition to its role in mediating a variety of antiproliferative processes in tumors, p53 can directly control transcription of proapoptotic members of the Bcl-2 family, such as Bax, with the net effect of increasing the ratio of pro- to antiapoptotic Bcl-2 proteins, thereby favoring caspase activation [14].

Apoptosis is a genetically controlled process. The aim of this study is to evaluate possible differences in the expression levels of apoptosis-related mRNAs (p53, Bax, Bcl-2, and caspase-3) activated by MPP^+^ using real-time quantitative RT-PCR analysis and evaluate the differential expression of a specific dopaminergic marker tyrosine hydroxylase (TH) in undifferentiated and RA-differentiated SH-SY5Y cells, for the assessment of suitable and accessible dopaminergic cell models for PD research.

## 2. Materials and Methods

### 2.1. Cell Culture

SH-SY5Y human neuroblastoma cells were grown in 1 : 1 mixture of MEM and F12, supplemented with 10% heat-inactivated fetal bovine serum (FBS), 1 mM sodium pyruvate, 0.1 mM nonessential amino acid, 1.5 g/L sodium bicarbonate, 100 units/mL penicillin, and 100 *μ*g/mL streptomycin. All media and supplements were purchased from Gibco (Gaithersburg, MD, USA). Cells were maintained at 37°C in a humidified atmosphere of 5% CO_2_. Cells were separated into two groups: undifferentiated and differentiated cells. For neuronal differentiation of SH-SY5Y cells, all-*trans*-RA was added a day after plating at final concentration 10 *μ*M in MEM-F12 with 10% FBS and maintained for 3 days. For MPP^+^ susceptibility experiment, undifferentiated and differentiated SH-SY5Y cells were plated at the same time and exposed to 125, 250, 500, 1000, and 2000 *μ*M of MPP^+^ for 24 hours. The control groups for undifferentiated and differentiated SH-SY5Y cells were treated with the same medium without MPP^+^.

### 2.2. MTT Assay

SH-SY5Y cells were seeded onto a 96-well plate at a density of 1 × 10^4^ cells/well in 200 *μ*L of medium and incubated at 37°C under 5% CO_2_. Cell viability was measured by MTT (3-(4,5-dimethylthiazol-2-yl)-2,5-diphenyltetrazolium bromide) colorimetric assay (Sigma-Aldrich, St. Louis, MO, USA). After incubation, 20 *μ*L of MTT (5 mg/mL) was added to each well and the cells were cultured for another 4 hours; then medium was removed and 100 *μ*L of DMSO was added to each well to dissolve the formazan. The color reaction, generated by the reduction of tetra ring of MTT by mitochondrial dehydrogenases with NADH in the active mitochondria, was measured spectrophotometrically at wavelength 570 nm with a reference at 665 nm using the VERSAmax Tunable microplate reader with SoftMax Pro software (Molecular Devices, Sunnyvale, CA, USA).

### 2.3. Western Blot Analysis for Tyrosine Hydroxylase

SH-SY5Y cells were grown on 6-well culture plates at a density of 2.5 × 10^4^ cells/well. After 24 hours, cells were treated with 10 *μ*M of RA in the basal medium. The medium was changed every 2 days. Protein was extracted on day 4, day 7, and day 10 with cold RIPA buffer (50 mM Tris pH 7.4, 150 mM NaCl, 1% triton X-100, 0.1% SDS, 1% sodium deoxycholate, 5 mM EDTA, 30 mM Na_2_HPO_4_, and 50 mM NaF). SH-SY5Y cells were immediately scraped off followed by centrifugation at 12,000 rpm for 15 minutes at 4°C. The supernatants were collected and protein concentrations were determined using Bradford reagent (Bio-Rad Laboratories, USA) by measuring absorbance at 595 nm. Equal amount of the protein was loaded onto 12.5% SDS-PAGE with a total volume of 20 *μ*L/well. The protein from the acrylamide gel was transferred to a nitrocellulose membrane. The membrane was blocked with skim milk in TBS-T at 4°C overnight and then incubated with primary rabbit polyclonal antibody to TH (1 : 1000 dilution; Cell Signal Technology) overnight. Subsequently, the membrane was incubated with horseradish peroxidase-conjugated goat anti-rabbit secondary antibody (1 : 5000 dilution) for 45 minutes at room temperature. Band intensity was determined using chemiluminescent (ECL) reagents (Pierce, USA) and analyzed on Image-J software (National Institutes of Health, Bethesda, MD, USA).

### 2.4. Immunostaining for Tyrosine Hydroxylase

SH-SY5Y cells were plated on 12 mm poly-L-lysine/laminin-coated coverslips at a density of 1 × 10^4^ cells/coverslip. Cells were treated with 10 *μ*M of RA for 4 and 7 days. Cells were then fixed in 4% paraformaldehyde for 15 minutes at room temperature followed by sequential incubation with permeabilizing solution (0.2% Triton X-100) in PBS for 30 minutes at room temperature. Then, cultures were washed again with PBS and incubated in blocking solution (3% BSA in 0.5% Tween 20 in PBS) for 30 minutes. Cells were incubated with rabbit polyclonal antibody against TH (1 : 200 dilution in blocking solution; Merck Millipore AB152) overnight at 4°C. After washing, cells were incubated with 1 : 500 dilution of Alexa 488-conjugate secondary antibody for 1 hour at room temperature. Coverslips were then mounted with Vectashield antifading mounting medium with DAPI (Vector Laboratories, CA). Cells were visualized under a confocal laser-scanning microscope (Olympus Model FV 1000, Tokyo, Japan).

### 2.5. Staining for Nuclear DNA

SY5Y cells were seeded on coverslips at density of 6 × 10^4^ cells/coverslip. For undifferentiated cells, cells were treated with 500 *μ*M MPP^+^ for 24 hours. For differentiated cells, 3-day treatment of 10 *μ*M RA was used for differentiation, and cells were treated with 1000 *μ*M MPP^+^ for 24 hours. Cells were washed, fixed with 4% paraformaldehyde for 30 minutes at room temperature, and washed with PBS. Cells were stained with Hoechst 33258 in PBS for 20 minutes at room temperature and then washed again. Coverslips were then mounted in 50% glycerol containing 20 mM citric acid and 50 mM orthophosphate. Nuclear morphology was visualized under a laser scanning confocal microscopy (Olympus FV1000, Olympus, Tokyo, Japan) with excitation wavelength 556 nm and emission wavelength 573 nm.

### 2.6. Real-Time Quantitative RT-PCR Analysis

Total mRNA was extracted from SH-SY5Y cells using PARIS kit according to the manufacturer's instructions. The quantity and purity of RNA were determined by optical density measurements at OD A260/A280 ratio with 1.8 or above using Nanodrop 2000 spectrophotometer (Thermo Fisher Scientific Inc., Wilmington, DE, USA). The cDNA was synthesized from 1 *μ*g of RNA using Masterscript RT-PCR System (5 PRIME, Gaithersburg, MD, USA), according to the manufacturer's instructions, and stored at −20°C until assay. KAPA SYBR FAST qPCR kit (Kapa Biosystems, Woburn, MA, USA) was used for real-time PCR quantification. The 20 *μ*L real-time PCR reaction mixture contained 20 ng cDNA template, 10 *μ*L of 1x KAPA SYBR FAST qPCR master mix, 200 nM of forward and reverse primers, and PCR-grade water. *β*-actin was used as a reference gene. The sequences of the primers for the RT-PCR were as follows: p53: sense, 5′-GGAGGTTGTGAGGCGCTGG-3′; antisense, 5′-CACGCACCTCAAAGCTGTTC-3′; Bax: sense, 5′-CCCGAGAGGTCTTTTTCCGAG-3′; antisense, 5′-CCAGCCCATGATGGTTCTGAT-3′; Bcl-2: sense, 5′-CATGTGTGTGGAGAGCGTCAA-3′; antisense, 5′-GCCGGTTCAGGTACTCAGTCA-3′; caspase-3: sense, 5′-ATGGAGAACACTGAAAACTCA-3′; antisense, 5′-TTAGTGATAAAAATAGAGTTC-3′; and *β*-actin: sense, 5′-TGCAGAGGATGATTGCTGAC-3′; antisense, 5′-GAGGACTCCAGCCACAAAGA-3′. The reaction was performed in the Applied Biosystems 7500 Real-Time PCR System (Applied Biosystems, Foster City, CA, USA) with the PCR cycling conditions as follows: 3-minute enzyme activation at 95°C, 40 cycles of 3-second initial denaturation at 95°C, and annealing/extension at 58°C for 32 seconds. Melting curve analysis was performed for verifying specificity of each primer after PCR to ensure amplification specificity. The threshold cycle (Ct) number was determined and used in the comparative Ct method. The relative quantity of the target gene was estimated by the 2^−ΔΔCt^ method. All data were analyzed by the ABI 7500 software, version 2.0.

### 2.7. Statistical Analysis

Experiments on cell viability assay, western blotting, and real-time quantitative PCR were repeated three times in triplicate measurement. Statistical analyses were performed with one-way ANOVA test followed by a post hoc analysis (Tukey's multiple comparison test) using GraphPad Prism 5 Software for Windows (GraphPad Software, Inc., San Diego, CA, USA). The results were expressed by mean ± standard error of the mean (mean ± SEM) for each group. A *P* valueless than 0.05 was regarded as statistically significant.

## 3. Results

### 3.1. Proliferation Rate of Undifferentiated and RA-Differentiated SH-SY5Y Cells

**Figure 1:**
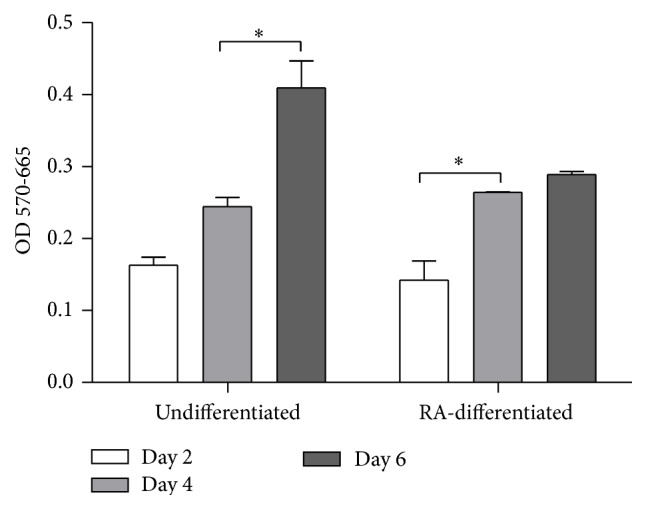
The proliferation of undifferentiated and differentiated SH-SY5Y cells. Cells were differentiated in 10 *μ*M retinoic acid (RA). After differentiation for 2, 4, and 6 days, cell proliferation was assessed using MTT assay. Absorbance at 570 nm with 665 nm as a reference was measured, and differences between the two absorbances were analyzed. Data are expressed as mean ± SD from three replicate wells. ^*∗*^ *P* < 0.05.

To study the effects of RA on the proliferation of SH-SY5Y cells, an MTT assay was performed to measure cell numbers. The MTT assay involves the use of mitochondrial activity in live cells to convert MTT to formazan, the concentration of which can be measured spectrophotometrically. The reduction of tetrazolium salts by metabolically active cells in the MTT assay is now widely accepted as a reliable way to examine cell proliferation. Absorbance values that are lower than the control cells indicate a reduction in the rate of cell proliferation. Conversely, a higher absorbance rate indicates an increase in cell proliferation. Statistically significant differences (*P* < 0.05) in cell number were observed between days 4 and 6 in undifferentiated cells and between days 2 and 4 in RA-differentiated cells (Figure 1). The proliferation rate was estimated as a percentage of the OD570-665 changes from day 2 to day 4 and from day 4 to day 6 for each group. In agreement with previous studies that have shown that cells treated with 10 *μ*M RA for 5 days cease proliferating and differentiate into a neuronal phenotype [15], our results showed a decline in the cell proliferation rate from day 4 to day 6 for RA-differentiated cells. In undifferentiated cells, the proliferation rate remained high from day 4 to day 6, compared to that from day 2 to day 4.

### 3.2. Expression of Tyrosine Hydroxylase Protein in Undifferentiated and RA-Differentiated SH-SY5Y Cells

**Figure 2:**
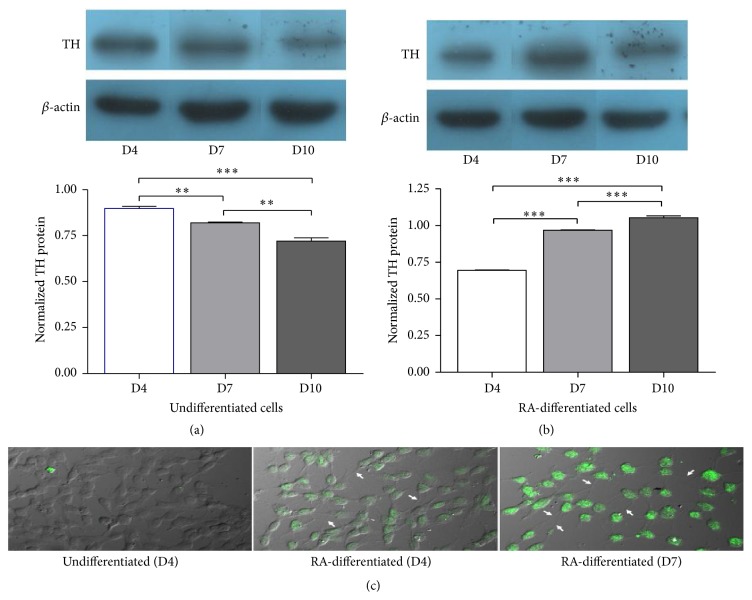
Expression of tyrosine hydroxylase in undifferentiated and differentiated SH-SY5Y cells. Cells were differentiated in 10-*μ*M retinoic acid (RA). After differentiation for 4, 7, and 10 days, tyrosine hydroxylase (TH) was detected with western blotting (a and b). The density of bands was analyzed in comparison with that of *β*-actin. Data are expressed as mean ± SEM (*n* = 3). ^*∗∗*^ *P* < 0.01; ^*∗∗∗*^ *P* < 0.001. Expression of TH was visualized through immunostaining in undifferentiated cells and 4- and 7-day RA-differentiated cells (c). White arrows indicate areas of neurite outgrowth.

To examine the effects of RA on changes in neuronal phenotypes, the expression of a dopaminergic neuronal marker tyrosine hydroxylase was evaluated in undifferentiated and RA-differentiated cells by the western blot immunoassay. A significant, gradual increase in TH protein content was observed in RA-differentiated cells from day 4 of differentiation to day 7 and day 10 (*P* < 0.001 for all comparisons; Figure 2(b)). In undifferentiated cells, the expression of TH protein was gradually decreased, significantly, from day 4 to day 7 and day 10 (Figure 2(a)). Based on these data, the expression of TH protein was reevaluated through immunostaining in undifferentiated cells and 4- and 7-day RA-differentiated cells (Figure 2(c)). The result confirmed a gradual increase in TH protein expression in differentiated SH-SY5Y cells along with the presence of neuritic outgrowth.

### 3.3. Susceptibility of Undifferentiated and RA-Differentiated SH-SY5Y Cells Exposed to MPP^+^

**Figure 3:**
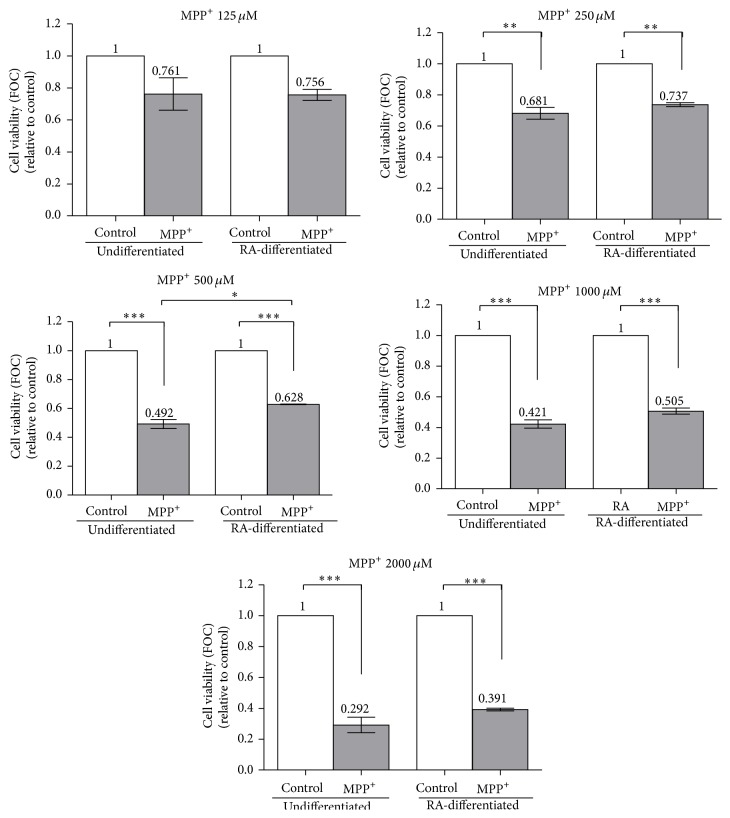
Effect of MPP^+^ on cell viability of undifferentiated and differentiated SH-SY5Y cells. Cells were differentiated in 10-*μ*M retinoic acid (RA) for 3 days. Undifferentiated and RA-differentiated cells were plated at the same time and exposed to 125, 250, 500, 1000, and 2000 *μ*M of MPP^+^ for 24 hours. Cell viability was examined using MTT assay. Data are expressed as fold of changes (FOC; mean ± SEM), relative to control (*n* = 3). ^*∗*^ *P* < 0.05; ^*∗∗*^ *P* < 0.01; and ^*∗∗∗*^ *P* < 0.001.

Undifferentiated and RA-differentiated SH-SY5Y neuroblastoma cells were exposed to 125, 250, 500, 1000, and 2000 *μ*M of MPP^+^ for 24 hours. For RA-differentiated cells, the cells were incubated with 10 *μ*M RA for 3 days to induce neuronal differentiation prior to exposure to the various dosages of MPP^+^. Cell viability was examined with an MTT assay. Significant decreases in cell viability of undifferentiated and RA-differentiated cells were observed after 24-hour exposure to 250 *μ*M of MPP^+^ when compared to the control (*P* < 0.01; Figure 3), and higher dosages of MPP^+^ led to higher numbers of cell death in both undifferentiated and RA-differentiated cells (*P* < 0.001). The different susceptibility of undifferentiated and RA-differentiated cells to MPP^+^ was observed at an MPP^+^ dosage of 500 *μ*M compared to untreated cells, and, after RA-differentiation, an MTT assay showed a 0.1-fold increase (*P* < 0.05) in the number of viable cells compared to undifferentiated cells 24 hours after exposure to MPP^+^. It is noteworthy that a decrease in cell viability to approximately 50% (IC_50_) required 500 *μ*M and 1000 *μ*M of MPP^+^ for undifferentiated and RA-differentiated cells, respectively. These dosages of MPP^+^ were used to treat undifferentiated and RA-differentiated cells in further experiments.

**Figure 4:**
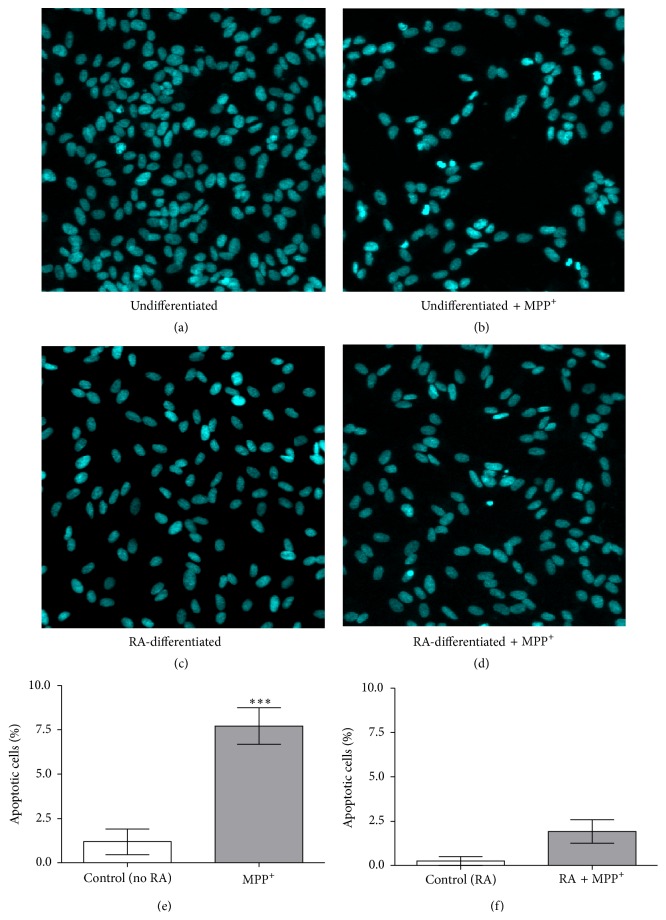
Effect of MPP^+^ on nuclear morphology in undifferentiated and differentiated SH-SY5Y cells. Cells were undifferentiated (a and b) and were differentiated in 10-*μ*M retinoic acid (RA) for 3 days (c and d). Undifferentiated (b) and differentiated cells (d) were exposed to 500 and 1000 *μ*M of MPP^+^ for 24 hours, respectively. Apoptotic nuclear morphology was visualized by DNA staining with Hoechst 33258. Percentage of cells with apoptotic nuclei was calculated (e and f). Data are expressed as mean ± SEM (*n* = 3) of percentage to untreated cells. ^*∗∗∗*^ *P* < 0.001.

Nuclear morphology of undifferentiated and RA-differentiated cells was further examined using Hoechst 33258 staining after exposure of the cells to 500 and 1000 *μ*M MPP^+^ for 24 hours, respectively. After treatment with 500 MPP^+^, undifferentiated cells showed distinct morphological changes typical of apoptosis, such as chromatin condensation and nuclear fragmentation (Figure 4(b)). Similar changes in nuclear morphology were also observed in RA-differentiated cells treated with 1000 *μ*M MPP^+^ (Figure 4(d)). Apoptotic nuclei were significantly observed in MPP^+^-treated undifferentiated cells (*P* < 0.001; Figures 4(e) and 4(f)). It is worth noting that the degree of apoptotic nuclei observed in Hoechst staining did not correspond to the degree of cell death observed in the MTT assay (Figure 3). More detailed studies are required to examine this apparent irrelevance. Given our culture conditions being constant and chemicals preparation being unchanged, one hypothesis is that the MTT assay may detect changes in the number of metabolically active cells, which occur earlier than changes in DNA morphology.

### 3.4. Expression of Apoptosis-Related Genes in Undifferentiated and RA-Differentiated SH-SY5Y after Treatment with MPP^+^

**Figure 5:**
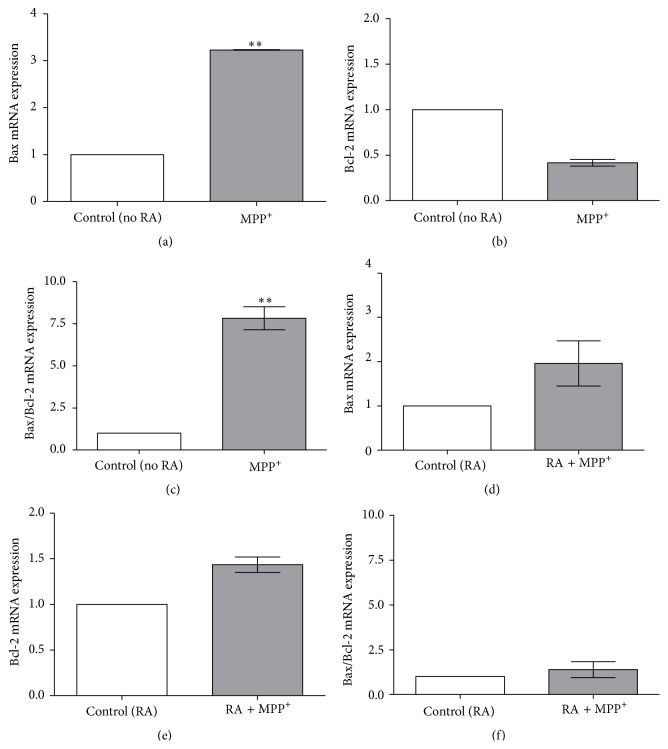
Effect of MPP^+^ on Bax and Bcl-2 mRNA expression in undifferentiated and differentiated SH-SY5Y cells. Cells were differentiated in 10-*μ*M retinoic acid (RA) for 3 days. Undifferentiated (a–c) and differentiated cells (d–f) were exposed to 500 and 1000 *μ*M of MPP^+^ for 24 hours, respectively. Expression of Bax mRNA (a and d), Bcl-2 mRNA (b and e), and Bax/Bcl-2 ratio (c and f) was analyzed with quantitative real-time RT-PCR, relatively compared to their respective untreated controls. The expression levels of the target gene were normalized to the expression level of *β*-actin. Data are expressed as mean ± SEM (*n* = 3). ^*∗∗*^ *P* < 0.01.

**Figure 6:**
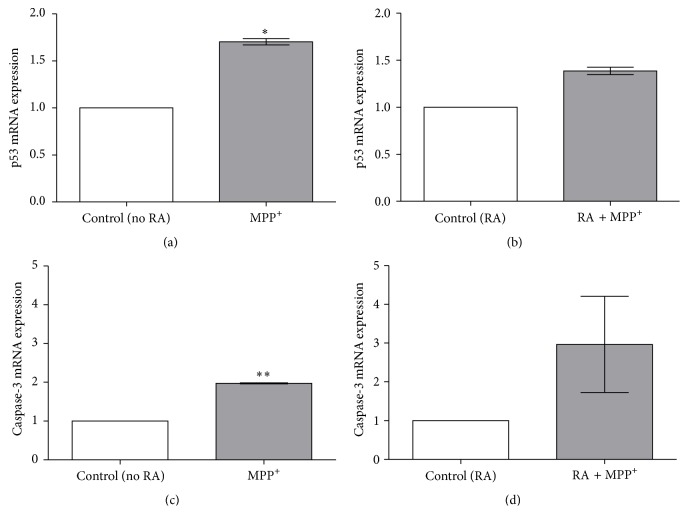
Effect of MPP^+^ on p53 and caspase-3 mRNA expression in undifferentiated and differentiated SH-SY5Y cells. Cells were differentiated in 10-*μ*M retinoic acid (RA) for 3 days. Undifferentiated (a and c) and differentiated cells (b and d) were exposed to 500 and 1000 *μ*M of MPP^+^ for 24 hours, respectively. Expression of p53 mRNA (a and b) and caspase-3 (c and d) was analyzed with quantitative real-time RT-PCR, relatively compared to their respective untreated controls. The expression levels of the target gene were normalized to the expression level of *β*-actin. Data are expressed as mean ± SEM (*n* = 3). ^*∗*^ *P* < 0.05; ^*∗∗*^ *P* < 0.01.

To observe the expression of apoptosis-related genes in SH-SY5Y cells that were exposed to IC_50_ dosages of MPP^+^, the genes Bax, Bcl-2, p53, and caspase-3 were tested using quantitative real-time RT-PCR analyses of 24-hour incubation of undifferentiated and RA-differentiated cells in 500 *μ*M and 1000 *μ*M MPP^+^, respectively. As Bax was increased by MPP^+^ whereas Bcl-2 was not, the Bax/Bcl-2 ratio was significantly increased by MPP^+^ in undifferentiated cells (*P* < 0.01; Figures 5(a)–5(c)). In RA-differentiated cells, both Bax and Bcl-2 were increased, leading to an insignificant change in the Bax/Bcl-2 ratio (Figures 5(d)–5(f)). An upstream regulation of Bax and Bcl-2 was then investigated. The expression of p53 mRNA was significantly increased in undifferentiated cells after 24-hour exposure to 500 *μ*M MPP^+^ when compared to the unexposed control (*P* < 0.05; Figure 6(a)), whereas it was not significantly increased in RA-differentiated cells exposed to 1000 *μ*M MPP^+^ (Figure 6(b)). Finally, the effect of MPP^+^ on the expression of caspase-3 mRNA, a critical executioner of apoptosis, was investigated. The expression of caspase-3 mRNA was significantly increased in undifferentiated cells treated with 500 *μ*M MPP^+^ for 24 hours when compared to the unexposed control (*P* < 0.01; Figure 6(c)), whereas it was not significantly increased in RA-differentiated cells treated with 1000 *μ*M MPP^+^ (Figure 6(d)).

## 4. Discussion

The human neuroblastoma SH-SY5Y cell line has been widely used in PD research. The question of whether undifferentiated or differentiated SH-SY5Y cells are more suitable remains controversial. The differentiated phenotype of SH-SY5Y cells is claimed to be more appropriate due to expressing desired morphological and biochemical characteristics similar to dopaminergic neurons and its lack of oncogenic and mitogenic properties [3]. However, changes in the dopaminergic properties of differentiated cells were found not to be significant compared to undifferentiated cells, and use of RA as an inducing agent may modulate the Akt prosurvival pathway leading to a higher tolerance to neurotoxin toxicity [2].

To address this unresolved issue, the differential expression of a specific marker of dopaminergic neurons and the differential response in the expression of apoptosis-related genes to a neurotoxin MPP^+^ in undifferentiated and RA-differentiated SH-SY5Y cells were assessed. First, the expression of TH protein could be detected in undifferentiated cells, and the expression was gradually decreased in relation to the longer culture duration, whereas the expression of TH protein was gradually increased in RA-differentiated cells from day 4 to at least day 10. Second, undifferentiated cells required a lower MPP^+^ dosage (500 *μ*M) than RA-differentiated cells (1000 *μ*M) to cause a 50% cell death, and apoptotic nuclei could be more significantly observed in 500-*μ*M MPP^+^-treated undifferentiated cells than in the 1000-*μ*M MPP^+^-treated differentiated cells, compared to their untreated controls. Lastly, treatment with 500 *μ*M MPP^+^ led to significant increases in the Bax/Bcl-2 ratio, p53, and caspase-3 mRNA expression in undifferentiated cells, whereas a significant increase was not observed in differentiated cells treated with 1000 *μ*M MPP^+^. In our study on cell susceptibility to MPP^+^ and the expression of apoptosis-related genes, a 3-day duration of 10 *μ*M RA exposure was used to induce SH-SY5Y cells for neuronal differentiation, which is based on our observations and those of other studies showing that TH expression and neurite outgrowth can be observed at this early period (Figures 2(b) and 2(c)) [16–19].

The results of cell susceptibility to MPP^+^ and the level of expression of apoptosis-related genes suggested that differentiated cells are more resistant to the effects of MPP^+^. Cheung et al. [2] showed that RA-differentiated cells also expressed higher tolerance, as shown by the lower rate of the release of LDH, cell mortality, and caspase-3 activation when compared to undifferentiated cells. However, Lopes et al. [3] showed that SH-SY5Y cells become significantly more sensitive to a toxin during the differentiation process as shown by cell mortality assay and GI_50_ values of the toxin. In general, a cell line with more sensitivity to toxin is regarded as being suitable for the investigation of a toxin mechanism or disease pathogenesis. Both studies by Cheung et al. and Lopes et al. showed contrasting results in the vulnerability for differentiated SH-SY5Y cells to toxins. There is no data on differences in cell susceptibility to neurotoxins between undifferentiated SH-SY5Y cells, RA-differentiated cells, and primary dopaminergic mesencephalic neurons. The higher tolerance of RA-differentiated SH-SY5Y cells to neurotoxins may be explained by the role of RA in the activation of the Akt prosurvival pathway [2]. In the brain, neurotrophic factors secreted from surrounding astrocytes can promote neuronal survival and differentiation. Neurotrophic factors can activate Akt signaling in neurons. Thus, RA-induced Akt activation may mimic the astrocyte environment that surrounds neurons in the brain, thereby increasing the validity of the differentiated SH-SY5Y cell model.

The results on the positive expression of TH protein using western blot analysis and immunostaining in undifferentiated SH-SY5Y cells are relevant with Cheung et al. [2], while Lopes et al. showed undetectable or very low levels of TH expression [3]. Both Cheung et al. and Lopes et al. measured TH immunocontent in undifferentiated cells at about 7 days after plating. After RA treatment, Lopes et al. found a significant increase in TH expression in differentiated cells at days 4, 7, and 10 similar to our results (Figure 2(b)) whereas Cheung et al. measured the expression at day 7 and did not find any increment. These discrepancies may reflect the uneven dynamics of TH protein translation in both proliferating undifferentiated cells and differentiated cells, which may be influenced by various conditions such as the differences in culture media used, which in Lopes et al. was DMEM/Ham's F12, in Cheung et al. was MEM, and in this study was MEM/Ham's F12. Greater amounts of amino acids, vitamins, and other substances in Ham's F12 may affect SH-SY5Y cell growth and differentiation [20]. Although TH expression can be found in undifferentiated cells, these cells do not secrete dopamine [21]. TH becomes active in RA-differentiated cells and these cells have a dopaminergic-like phenotype [1]. These data imply that considering whether undifferentiated or differentiated SH-SY5Y cells are suitable as a cellular model of PD based exclusively on the data of TH expression levels shown in various studies may not be a proper approach.

A previous study showed that significant cell death of RA-differentiated cells was observed after 24 hours of exposure to 500 and 1000 *μ*M MPP^+^, and there was no apparent increase in the cell death by extending the exposure period to 48 hours [22]. For undifferentiated cells, studies showed that cell viability decreased to approximately 50% when cells were treated with 500 *μ*M MPP^+^ for 24 hours [7, 23, 24]. Similarly, we observed significant cell death after 24-hour exposure of RA-differentiated cells as well as of undifferentiated cells to 250, 500, 1000, and 2000 *μ*M MPP^+^. Higher IC_50_ of MPP^+^ in RA-differentiated cells as shown in this study may indicate that their tolerance to MPP^+^ toxicity is higher than that of undifferentiated cells. Thus, we further examined the expression of Bax, Bcl-2, p53, and caspase-3 mRNAs to reaffirm changes in cell susceptibility to MPP^+^-induced neurotoxicity. Although increases in the Bax/Bcl-2 ratio, p53, and caspase-3 expression were observed in both 500 *μ*M MPP^+^-treated undifferentiated and 1000 *μ*M MPP^+^-treated differentiated cells compared to their own controls, significant increases were also apparently shown in undifferentiated cells. Lower susceptibility of RA-differentiated SH-SY5Y cells to 1000 *μ*M MPP^+^ and 25 *μ*M 6-OHDA has been demonstrated in a previous study based on the measurement of LDH release, cell viability, and caspase-3 activity in SH-SY5Y cells after RA-differentiation for 7 days [2]. However, our results suggest that the higher tolerance of RA-differentiated cells to 1000 *μ*M MPP^+^ can be observed as early as 3 days of RA-differentiation.

Higher tolerance of RA-differentiated SH-SY5Y cells to MPP^+^ and 6-OHDA may be explained by the role of RA in the activation of the Akt prosurvival pathway [2]. Although it has been stated that neuroblastoma cells have to be differentiated for at least 7 days for experimentation [25], there is no evidence showing changes of cell susceptibility to neurotoxins when comparing between shorter and longer durations for differentiation. There is also no data on differences in cell susceptibility to neurotoxins between RA-differentiated SH-SY5Y cells and primary dopaminergic mesencephalic neurons. With the lack of these data, one might raise the question of what length of time of RA-induced differentiation would be appropriate to make differentiated cells mimic primary dopaminergic neurons. Prolonged RA treatment of SH-SY5Y cells for 14 days can lead to an increase in dopamine transporter protein (DAT) expression [26], which may result in increased sensitivity of RA-differentiated cells to MPP^+^. MPP^+^ toxicity enhanced by overexpression of DAT has been reported in* Drosophila* dopaminergic neurons [27]. Thus, if prolonged RA treatment leads to increased DAT activity beyond the basal activity of primary dopaminergic neurons, a shorter duration of RA treatment may be appropriate to induce SH-SY5Y cells for a dopaminergic model of PD.

In addition to the role of RA in the Akt activation, the number of mitochondria in differentiated SH-SY5Y cells may be a predisposing factor to toxin susceptibility. Indirect evidence may shed light on the effect of differentiation on the mitochondrial number in SH-SY5Y cells. An average number of mitochondria, 10–22 per cell, has been measured in undifferentiated SH-SY5Y cells [28]. A study of primary hippocampal neurons showed that the number of mitochondria increases during BDNF-induced differentiation and maturation [29]. In SH-SY5Y cells, BDNF responsiveness can be induced by RA [30]. Based on this evidence, it is possible that increased numbers of mitochondria in differentiated cells could make the cells less susceptible to MPP^+^ toxicity if we assume that the number of mitochondria could be increased in SH-SY5Y cells upon RA-differentiation. To date, there is no study on the difference in the number of mitochondria between undifferentiated and differentiated SH-SY5Y cells.

SH-SY5Y neuroblastoma cells are a useful model for studying the behavior of catecholaminergic (nondifferentiated) or dopaminergic (differentiated) neurons. However, they do not mimic the behavior of primary dopaminergic neurons; they are essentially oncogenic cells and may be used with all inherent limitations.

## 5. Conclusion

Selecting between undifferentiated and differentiated SH-SY5Y neuroblastoma cells as a suitable and accessible cell model for PD research still needs further investigation. Based on our results, differentiated cells may be more suitable, and the shorter duration of RA differentiation may make the SH-SY5Y cell model more accessible. Counteraction of the opposite effect between prolonged activation of the prosurvival pathway and increased DAT expression by RA during cell differentiation remains to be investigated prior to the conclusion of prolonged RA-differentiated SH-SY5Y cells as an experimental model of primary dopaminergic neurons.
